# A novel *ABCD1* gene mutation causes adrenomyeloneuropathy presenting with spastic paraplegia: A case report

**DOI:** 10.1097/MD.0000000000037874

**Published:** 2024-04-19

**Authors:** Jinxin Liu, Xin Wang, Di Huang, Yuna Qi, Lei Xu, Yankun Shao

**Affiliations:** a Department of Neurology, China-Japan Union Hospital of Jilin University, Changchun, China.

**Keywords:** ABCD1, adrenomyeloneuropathy, case report, gene mutation, X-linked adrenoleukodystrophy

## Abstract

**Rationale:**

X-linked adrenoleukodystrophy (X-ALD) is caused by mutations in the ABCD1 gene leading to very long chain fatty acid (VLCFA) accumulation. The disease demonstrates a spectrum of phenotypes including adrenomyeloneuropathy (AMN). We aimed to identify the genetic basis of disease in a patient presenting with AMN features in order to confirm the diagnosis, expand genetic knowledge of ABCD1 mutations, and elucidate potential genotype-phenotype associations to inform management.

**Patient concerns:**

A 29-year-old male presented with a 4-year history of progressive spastic paraplegia, weakness of lower limbs, fecal incontinence, sexual dysfunction, hyperreflexia, and positive Babinski and Chaddock signs.

**Diagnoses:**

Neuroimaging revealed brain white matter changes and spinal cord thinning. Significantly elevated levels of hexacosanoic acid (C26:0) and tetracosanoic acid (C24:0) suggested very long chain fatty acids (VLCFA) metabolism disruption. Genetic testing identified a novel hemizygous ABCD1 mutation c.249dupC (p.F83fs). These findings confirmed a diagnosis of X-linked ALD with an AMN phenotype.

**Interventions:**

The patient received dietary counseling to limit VLCFA intake. Monitoring for adrenal insufficiency and consideration of Lorenzo’s oil were advised. Genetic counseling and testing were offered to at-risk relatives.

**Outcomes:**

At present, the patient continues to experience progressive paraplegia. Adrenal function remains normal thus far without steroid replacement. Family members have undergone predictive testing.

**Lessons:**

This case expands the known mutation spectrum of ABCD1-linked X-ALD, providing insight into potential genotype-phenotype correlations. A thoughtful diagnostic approach integrating clinical, biochemical and genetic data facilitated diagnosis. Findings enabled genetic counseling for at-risk relatives regarding this X-linked disorder.

## 1. Introduction

X-linked adrenoleukodystrophy (X-ALD; OMIM:300100), a rare and complex peroxisomal disorder, is distinguished by progressive demyelination of the cerebral white matter, a gradual myeloneuropathy evolution, and irregularities in adrenal cortex function.^[1,2]^ It arises due to a deficiency in the ATP-binding cassette sub-family D member 1-a protein encoded by the ABCD1 gene, leading to impairment in the β-oxidation of very long-chain fatty acids (VLCFA). Consequently, an abnormal surge in VLCFA is observed within the plasma and tissues, with a particular inclination for the brain’s white matter, spinal cord, and adrenal cortex.^[3]^

According to the age of onset, the principal site of the central nervous system (CNS) lesions, and the pace of progress, X-ALD is divided into different phenotypes: adrenomyeloneuropathy (AMN), Addison-only, cerebellar variant, childhood cerebral ALD, adolescent cerebral ALD, adult cerebral ALD, asymptomatic, and presymptomatic female heterozygotes.^[4]^ AMN accounts for 40% of ALD cases,^[1]^ and it is the most common cause of metabolic hereditary spastic paraplegia in adults.^[5]^

Despite the numerous pathogenic variants in the ABCD1 gene listed in the ALD Mutation Database (last modified on 2023-12-06, https://adrenoleukodystrophy.info/mutations-and-variants-in-abcd1), the mutation highlighted in our study has not been previously reported. Our report focuses on a novel frameshift variant in the ABCD1 gene, observed in a 29-year-old Chinese male diagnosed with ALD and presenting as AMN. This case report follows CARE guidelines (for CAse REports) (Supplementary Guidelines Checklist, http://links.lww.com/MD/M225).

## 2. Case presentation

The patient granted permission to the authors and their institution to use his anonymized medical information to publish this case report in a medical journal. Written consent for publication was obtained from the patient. According to the regulations of the authors’ institution, the informed consent of the patient is required to publish the case report, and the approval of the ethics committee is not necessary.

A 29-year-old male presented with a 4-year history of progressive spastic paraplegia. The patient’s vital signs were within normal range. Initial neurological physical examination revealed diminished muscle strength (grade 3) in both lower extremities, increased abdominal reflex, absent right cremasteric reflex and anal reflex, hyperactive tendon reflexes in all limbs, positive bilateral Babinski and Chaddock signs. He suffered from fecal incontinence twice and sexual dysfunction. Romberg signs could not be checked because the patient was not able to stand with feet together without assistance for several seconds. He had a spastic gait, dragging both feet and unable to walk 10 meters. Physical examination revealed intact motor function in both upper limbs and normal sensory function in the whole body. Both ankle and patellar clonus were absent bilaterally. Cranial nerves I-XII were grossly intact on testing. No orthostatic hypotension was elicited in recumbent-upright test. The clinical characteristics of the patient are summarized in Table 1. We obtained the publicly available Functional Systems Scores (FSS) and Expanded Disability Status Scale (EDSS) materials from the National Multiple Sclerosis Society website (https://www.nationalmssociety.org/).^[6]^ Using these tools, we evaluated a patient with an EDSS score of 3.5. The detailed FSS and EDSS results for this patient are presented in Table S1, Supplemental Digital Content, http://links.lww.com/MD/M227.

**Table 1:** Clinical characteristics of the patient.

Gender	Male	Abdominal reflex (L/R)	+++/+++
Age of diagnosis (year)	29	Cremasteric reflex (L/R)	++/0
Age of onset (year)	25	Anal reflex	0
Initial symptoms	Weakness of lower limbs	Tendon reflexes (UL/LL)	+++/+++
Spinal symptoms	Spastic paraplegia	Babinski sign	Positive
Peripheral neuropathy	No	Chaddock signs	Positive
Cognitive impairment	No	Sensory	Normal
Sphincter dysfunction	Yes	Ataxia	No
Hypoadrenocorticism	No	Cerebral involvement in MRI	Yes
Sexual dysfunction	Yes	Spinal involvement in MRI	Yes
Muscle strength (UL/LL)	5/3	Disease progression	Slow progression
Muscle tension (UL/LL)	Increased/Increased		

+ *=* decreased reflex, ++ *=* normal reflex, +++ *=* brisk reflex, ++++ *= hyperreflexi*a, 0 *=* areflexia*, L/R =* left/right, UL/LL *=* upper limbs/lower limbs.

Routine hematological and biochemical tests were unremarkable and adrenal function remained within regular parameters. The patient also tested within standard ranges for infectious and autoimmune markers, including hepatitis, HIV (Human immunodeficiency virus), treponema pallidum, anti-cardiolipin antibody, immunoglobulins, complement, anti-CCP (Anti- cyclic citrullinated peptide), ANA (anti-nuclear antibody), ANCA (anti-neutrophil cytoplasmic antibodies), RF (rheumatoid factor), ASO (anti-streptolysin O). Furthermore, Vitamin B12 levels were confirmed to be in the normal range.

Neuroimaging displayed slight gyral atrophy in bilateral parietal lobes and minor demyelination around the posterior horns of lateral ventricles on head MRI (Magnetic resonance imaging), and thinning of the thoracic and cervical spinal cord on spinal MRI (Figure S1, Supplemental Digital Content, http://links.lww.com/MD/M226). No abnormalities were observed on adrenal gland CT (computed tomography).

Based on the initial signs and symptoms, the MRI results of the brain and spinal cord, and a comprehensive yet unremarkable battery of autoimmune, infectious, inflammatory, vascular, endocrine, and nutritional evaluations, the differential diagnosis narrowed down to ALD presenting as AMN and hereditary spastic paraplegia. Consequently, we proceeded with serum VLCFA level assessment and genetic testing.

**Figure 1.**
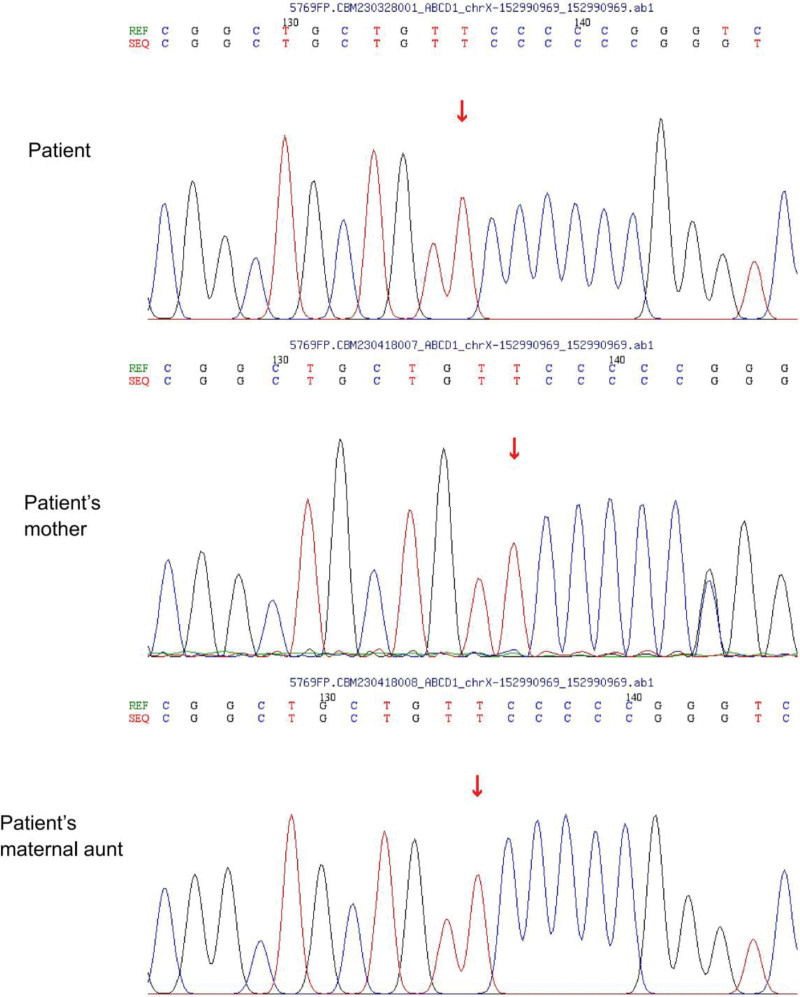
Sanger sequencing result for the c.249dupC mutation site of the patient, patient’ s mother and patient’ s maternal aunt. The numbers 130 and 140 are just identifiers for the lab automation workflow and have no specific meaning.

Significant elevations of hexacosanoic acid (C26:0), tetracosanoic acid (C24:0) levels and C24/C22, C26/C22 suggested a disorder in peroxisomal very long-chain fatty acid metabolism (Table S2, Supplemental Digital Content, http://links.lww.com/MD/M228). Whole exome sequencing revealed a pathogenic hemizygous mutation (c.249dupC) in the ABCD1 gene, which was linked to X-linked adrenoleukodystrophy (Fig. 1). We identified a novel hemizygous frameshift mutation, c.249dupC, in the ABCD1 gene (NM_000033). This duplication of a cytosine at codon 249, as indicated by the arrows in the Figure 1, resulted in a frameshift starting at the 83rd amino acid position, changing phenylalanine to a frameshifted sequence (p.F83fs) in exon 1.The chromosomal location was chrX:152990969 (GRCh37). This mutation followed an X-linked recessive inheritance pattern and was classified as Pathogenic (PVS1 + PS1 + PM2) according to the American College of Medical Genetics and Genomics guidelines. Its frequency in the normal population was 0. This variant was not registered in the ALD Mutation Database and gnomAD v2.1.1 (http://www.gnomad-sg.org/).

To delve deeper into the genetic etiology of this condition, we employed Sanger sequencing (Fig. 1) on the same gene segments (chrX:152990969) derived from the patient’s mother and maternal aunt. This genetic examination unveiled a heterozygous mutation (c.249dupC, p.F83fs) within the gene segment in the patient’s mother, whilst his aunt displayed no such mutation. Despite the presence of this mutation, his mother exhibited no discernible symptoms or abnormal sign, and his aunt, of course, did not show any signs of physical abnormality.

Following the confirmed diagnosis of X-ALD (AMN), dietary advice was given to the patient to favor foods high in unsaturated fatty acids and limit those with very long-chain fatty acids. Lorenzo’s oil was suggested as a treatment to normalize VLCFAs in the brain and potentially decelerate ALD progression.^[7]^ A neurological follow-up was scheduled within 3-6 months, with corticosteroid replacement therapy to be considered if serum corticosteroid levels are found to be low.

## 3. Discussion and conclusions

X-linked adrenoleukodystrophy (X-ALD) is a complex and heterogeneous disease caused by mutations in the ABCD1 gene, manifesting in a variety of phenotypes, one of which is adrenomyeloneuropathy (AMN). AMN manifests clinically in affected males most commonly in their third or fourth decade of life, with a mean age at symptom onset of 27.6 years.^[8]^ We herein report a novel ABCD1 mutation presenting as AMN in a 29-year-old male patient. This adds to the growing knowledge of the genetic basis of this disorder and provides further insight into the possible genotype-phenotype correlations in X-ALD. Future research should aim to find more novel gene mutations in ABCD1 and to elucidate the association between genotypes and phenotypes in ALD.

From a biochemical perspective, there is an accumulation of very long-chain fatty acids along with a partial disruption of peroxisomal oxidation in X-ALD patients.^[9]^ Cells derive energy from fatty acids, which undergo oxidation. This process is initiated by acyl-CoA synthetase in the cytosol, converting these fatty acids into fatty acyl-coenzyme A (fatty acyl-CoA). Very long-chain (>C20) fatty acyl-CoAs (VLCFA-CoAs) are then transported into peroxisomes by ATP-binding cassette (ABC) transporters. These transporters utilize the energy from ATP breakdown. In X-ALD patients, pathogenic variants in the ABCD1 gene hinder the entry of VLCFA-CoAs into the peroxisome for β-oxidation. This gene encodes the ABC transporter ABCD1, located in the peroxisomal membrane. Due to this obstruction, VLCFA-CoAs accumulate in the cytosol, adversely impacting the stability of the adrenal gland, testis, and myelin.^[10,11]^ This mutation induces a frameshift at codon 83, likely resulting in the premature termination of the codon and the subsequent production of a truncated, nonfunctional ABCD1 protein that lacks most of its transmembrane domains and ATP-binding domain. In addition, this mutation may also lead to breakdown of conformational states of ABCD1 protein.^[12]^

Institutions in some places (e.g., New York State in the US) were allowed to conduct newborn screening for ALD disease prior to development of clinical manifestations and offer the opportunity for further genetic characterization and phenotyping, which showed the importance of identifying new gene mutation sites and corresponding phenotypes.^[13,14]^ A novel hemizygous frameshift mutation c.249dupC (p.F83fs) in exon1 of the ABCD1 gene was identified in this case. This duplication mutation manifested as an extra C nucleotide inserted after the T nucleotide indicated by the arrow in Figure 1. Since there were normally 5 C nucleotides following this T in the reference sequence, the overlapping peak (heteroduplex peak) was only observed at the 6th nucleotide position in the electropherogram (see the sequencing result for the patient’s mother in Figure 1 for comparison). As the patient was male with only one X chromosome, this was a hemizygous variant and no overlapping peaks were seen in his DNA sequencing results. This novel mutation sites filled gaps in the map of pathogenic mutations in the ABCD1 gene, and phenotypic presentation associated with the novel mutation described here was compared to phenotypes linked to other reported mutations occurring at adjacent sites, as summarized in Table 2.

**Table 2:** Reported mutations adjacent to the novel mutation sites described in this study, and associated phenotypes.

Number	Chromosome position	ABCD1 mutation site	Mutation type	Consequence	Exon	Phenotype	Reference
1	152990961	c.240_41insTTTGCG	insertion	p.Arg80_Leu81insPheAla	1	AMN	^[15]^
2	152990961	c.240_41insTCCTGCGGC	insertion	p.Arg80_Leu81insSerCysGly	1	cALD	^[16]^
3	152990964	c.244_245insCTGCGGCTC	insertion	p.Leu81_Leu82insProAlaAla	1	Not classified	^[17]^
4	152990969	c.248del	deletion	p.Phe83Serfs*20	1	Unknown	Unpublished data
5	152990969	c.249dupC	duplication	p.F83fs	1	AMN	This article
6	152990971	c.250C > T	missense	p.Pro84Ser	1	AMN	^[18]^
7	152990972	c.251C > T	missense	p.Pro84Leu	1	AMN	^[19]^
8	152990974	c.253dup	insertion	p.Arg85Profs*110	1	AMN	^[20]^
9	152990974	c.253del	deletion	p.Arg85Glyfs*18	1	cALD	^[21]^
10	152990975	c.254_900 + 760del	deletion	p.Val86Glyfs*99	1	Not classified	^[22]^
11	152990978	c.257_268dup	duplication	p.Val86_Arg89dup	1	cALD	^[23]^

cALD = cerebral adrenoleukodystrophy.

The ABCD1 gene, which is instrumental in the pathogenesis of X-ALD, is located on the long arm of chromosome X (Xq28). When a pathogenic mutation occurs on the ABCD1 gene, this variation can be transmitted to subsequent generations through the X chromosome, illustrating the X-linked inheritance of this disorder. This gene mutation manifests with a high penetrance rate, reaching 100% in males and close to 65% in heterozygous females by the age of 60 years.^[24]^ Consequently, X-ALD is classified as one of the most common monogenetically inherited neurodegenerative diseases, affecting about 1 to 5 in 100,000 people worldwide,^[25]^ which underlines its clinical significance and the urgency for further research into its pathogenesis, including genotype-phenotype correlations. Definitively establishing genotype-phenotype correlations in X-ALD remains challenging given the complex multifactorial nature of the disease pathogenesis. While ABCD1 mutations are implicated, additional genetic and non-genetic factors such as trauma, epigenetic modifications, and environmental exposures may contribute to or modify disease onset and progression.^[4]^

X-ALD can present as various phenotypes, among which adrenomyeloneuropathy (AMN) is a common manifestation. The symptoms of AMN predominantly involve the nervous system and can include slowly progressive spastic paraparesis, sensory ataxia, and dysfunctions of the bowel and bladder, occasionally accompanied by fecal incontinence.^[26]^ Our patient demonstrated a clinical presentation that is typical for AMN. This included weakness of lower limbs,^[27]^ progressive spastic paraplegia, hyperactive tendon reflexes, and positive pathologic reflexes, along with fecal incontinence. These clinical features were indicative of a central nervous system lesion, which were corroborated by neuroimaging data. However, He did not show signs of adrenal insufficiency, sensory impairment and bladder dysfunction, which are also possible features of AMN.^[13,28]^

Therefore, X-ALD diagnosis poses significant challenges due to its diverse presentation and similarity to other neurological diseases such as hereditary spastic paraplegia and Schilder disease. Given these challenges, comprehensive and thoughtful diagnostic testing, involving neuroimaging, biochemical assays, and genetic testing, is crucial. In our case, significant elevations in VLCFA levels and a novel mutation in ABCD1 detected by whole exome sequencing provided the necessary diagnostic clarity.

**Figure 2.**
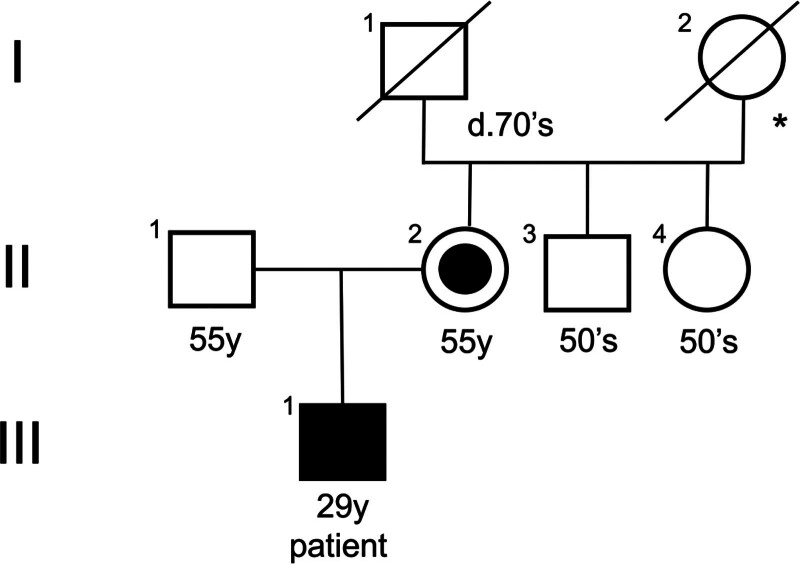
Genogram. *Unknown age of death.

It is also important to recognize the implications of this diagnosis on a familial level (Fig. 2), given its X-linked recessive inheritance. The discovery of the same mutation in the patient’s mother underlines the genetic risk for potential offspring. Although the patient’s aunt tested negative for the mutation, genetic counseling should still be offered to all at-risk relatives. Counseling can help family members understand their risk, the implications of the mutation, and the options available for predictive genetic testing.

In terms of X-ALD management, there is no cure for X-ALD for the time being. Our treatment approach centered around symptomatic management, with dietary modifications and monitoring for adrenal insufficiency. Lorenzo’s oil, a blend of unsaturated fatty acids that hinders the elongation of saturated fatty acids, primarily benefits presymptomatic patients, having unfortunately limited efficacy when applied post-disease onset.^[29]^ The aberrant adrenal response to ACTH (adrenocorticotropic hormone) stimulation seen in X-ALD arises from an increase in the microviscosity of the adrenocortical cell membrane. This is instigated by the accumulation and integration of VLCFAs into the phospholipid cell membrane, leading to a gradual breakdown of membrane functions.^[30]^ Timely steroid replacement therapy is imperative for AMN patients with borderline adrenal function (baseline plasma ACTH > 100 pg/mL, morning fasting cortisol level > 6 μg/dL, normal cortisol response to ACTH stimulation), due to the risk of acute adrenal failure.^[31]^ Patients with hypoadrenal functionality necessitate steroid supplementation. Additionally, bone marrow transplantation emerges as an efficacious long-term treatment, albeit exclusively for selected childhood cerebral X-ALD patients in early disease stages.^[32]^

While this case report expands the known genetic spectrum of X-ALD and AMN phenotype associations, our study has some limitations. As a single case report, our findings have limited generalizability. Additional cases demonstrating this novel ABCD1 mutation will be needed to further define potential genotype-phenotype correlations. Our phenotypic characterization is also limited by the lack of longitudinal follow-up data. The natural progression of symptoms and adrenal dysfunction could not be delineated. Additionally, our analysis did not include functional assays to directly assess effects of this mutation on encoded protein and enzymatic function. In vitro studies could elucidate consequences on ABCD1 expression, very long chain fatty acid transport and metabolism. Finally, the potential contribution of modifier genes and environmental factors cannot be excluded.^[33,34]^ Variants in other genes implicated in peroxisomal pathways may combine with this ABCD1 mutation to influence disease presentation and progression. A multi-omics approach could shed light on additional genetic contributors. Further analyses in expanded patient cohorts, with serial long-term follow-up and mechanistic assays, can build upon these preliminary case findings. Additional research is essential to determine predictive values for genotypes in X-ALD, given challenges in clear-cut phenotype delineation and anticipation of disease course.

In conclusion, we identified a novel frameshift variant in the ABCD1 gene in a Chinese patient with X-ALD who presented with adrenomyeloneuropathy. Our findings enrich the genetic landscape of X-ALD and underscore the value of integrating clinical, biochemical, and especially genetic data for an accurate diagnosis. This case augments our comprehension of the phenotypic heterogeneity associated with ABCD1 mutations in X-ALD, and provide a foundation for future studies of potential genotype-phenotype correlations involving mutations in this gene. Findings enabled genetic counseling for at-risk relatives regarding this X-linked disorder.

## Acknowledgments

We thank the patient and his family for their active cooperation.

## Author contributions

**Conceptualization:** Jinxin Liu, Yankun Shao.

**Data curation:** Jinxin Liu, Xin Wang, Di Huang, Yuna Qi.

**Writing – original draft:** Jinxin Liu.

**Writing – review & editing:** Lei Xu, Yankun Shao.

## Supplementary Material








