# Supplementary material for: A novel ABCD1 gene mutation causes adrenomyeloneuropathy presenting with spastic paraplegia: A case report
Source: Medicine (Baltimore). 2024 Apr 19;103(16):e37874. doi: 10.1097/MD.0000000000037874 (PMC11029984; doi:10.1097/MD.0000000000037874)
Supplement: Supplementary file 2 [file medi-103-e37874-s002.pdf]

Supplementary Table S1. FSS and EDSS results for the patient.

Kurtzke Functional Systems Scores (FSS)

❑ **Pyramidal Functions** Score: 3

0 - Normal
1 - Abnormal signs without disability
2 - Minimal disability
3 - Mild to moderate paraparesis or hemiparesis (detectable weakness but most function sustained for short periods, fatigue a problem); severe monoparesis (almost no function)
4 - Marked paraparesis or hemiparesis (function is difficult), moderate quadriparesis (function is decreased but can be sustained for short periods); or monoplegia
5 - Paraplegia, hemiplegia, or marked quadriparesis
6 - Quadriplegia
9 - (Unknown)

❑ **Cerebellar Functions** Score: 3

0 - Normal
1 - Abnormal signs without disability
2 - Mild ataxia (tremor or clumsy movements easily seen, minor interference with function)
3 - Moderate truncal or limb ataxia (tremor or clumsy movements interfere with function in all shpheres)
4 - Severe ataxia in all limbs (most function is very difficult)
5 - Unable to perform coordinated movements due to ataxia
9 - (Unknown)

❑ Record #1 in small box when weakness (grade 3 or worse on pyramidal) interferes with testing.

❑ **Brainstem Functions** Score: 0

0 - Normal
1 - Signs only
2 - Moderate nystagmus or other mild disability
3 - Severe nystagmus, marked extraocular weakness, or moderate disability of other cranial nerves
4 - Marked dysarthria or other marked disability
5 - Inability to swallow or speak
9 - (Unknown)

❑ **Sensory Function** Score: 0

0 - Normal
1 - Vibration or figure-writing decrease only in one or two limbs
2 - Mild decrease in touch or pain or position sense, and/or moderate decrease in vibration in one or two limbs; or vibratory (c/s figure writing) decrease alone in three or four limbs
3 - Moderate decrease in touch or pain or position sense, and/or essentially lost vibration in one or two limbs; or mild decrease in touch or pain and/or moderate decrease in all proprioceptive tests in three or four limbs
4 - Marked decrease in touch or pain or loss of proprioception, alone or combined, in one or two limbs; or moderate decrease in touch or pain and/or severe proprioceptive decrease in more than two limbs
5 - Loss (essentially) of sensation in one or two limbs; or moderate decrease in touch or pain and/or loss of proprioception for most of the body below the head
6 - Sensation essentially lost below the head
9 - (Unknown)

❑ **Bowel and Bladder Function** Score: 0

(Rate on the basis of the worse function, either bowel or bladder)
0 - Normal
1 - Mild urinary hesitance, urgency, or retention
2 - Moderate hesitance, urgency, retention of bowel or bladder, or rare urinary incontinence (intermittent self-catheterization, manual compression to evacuate bladder, or finger evacuation of stool)
3 - Frequent urinary incontinence
4 - In need of almost constant catheterization (and constant use of measures to evacuate stool)
5 - Loss of bladder function
6 - Loss of bowel and bladder function
9 - (Unknown)

❑ **Visual Function** Score: 0

0 - Normal
1 - Scotoma with visual acuity (corrected) better than 20/30
2 - Worse eye with scotoma with maximal visual acuity (corrected) of 20/30–20/59
3 - Worse eye with large scotoma, or moderate decrease in fields, but with maximal visual acuity (corrected) of 20/60–20/99
4 - Worse eye with marked decrease of fields and maximal visual acuity (corrected) of 20/100–20/200; grade 3 plus maximal acuity of better eye of 20/60 or less
5 - Worse eye with maximal visual acuity (corrected) less than 20/200; grade 4 plus maximal acuity of better eye of 20/60 or less
6 - Grade 5 plus maximal visual acuity of better eye of 20/60 or less
9 - (Unknown)

❑ Record #1 in small box for presence of temporal pallor

❑ **Cerebral (or Mental) Functions** Score: 0

0 - Normal
1 - Mood alteration only (does not affect EDSS score)
2 - Mild decrease in mentation
3 - Moderate decrease in mentation
4 - Marked decrease in mentation (chronic brain syndrome – moderate)
5 - Dementia or chronic brain syndrome – severe or incompetent
9 - (Unknown)

Sources: Kurtzke JF. Rating neurologic impairment in multiple sclerosis: an expanded disability status scale (EDSS). Neurology. 1983 Nov;33(11):1444-52.

Haber A, LaRocca NG. eds. Minimal Record of Disability for multiple sclerosis. New York: National Multiple Sclerosis Society; 1985.

Kurtzke Expanded Disability Status Scale (EDSS)

Score: 3.5

- ❑ 0.0 - Normal neurological exam (all grade 0 in all Functional System (FS) scores*).
- ❑ 1.0 - No disability, minimal signs in one FS* (i.e., grade 1).
- ❑ 1.5 - No disability, minimal signs in more than one FS* (more than 1 FS grade 1).
- ❑ 2.0 - Minimal disability in one FS (one FS grade 2, others 0 or 1).
- ❑ 2.5 - Minimal disability in two FS (two FS grade 2, others 0 or 1).
- ❑ 3.0 - Moderate disability in one FS (one FS grade 3, others 0 or 1) or mild disability in three or four FS (three or four FS grade 2, others 0 or 1) though fully ambulatory.
- ❑ 3.5 - Fully ambulatory but with moderate disability in one FS (one grade 3) and one or two FS grade 2; or two FS grade 3 (others 0 or 1) or five grade 2 (others 0 or 1).
- ❑ 4.0 - Fully ambulatory without aid, self-sufficient, up and about some 12 hours a day despite relatively severe disability consisting of one FS grade 4 (others 0 or 1), or combination of lesser grades exceeding limits of previous steps; able to walk without aid or rest some 500 meters.
- ❑ 4.5 - Fully ambulatory without aid, up and about much of the day, able to work a full day, may otherwise have some limitation of full activity or require minimal assistance; characterized by relatively severe disability usually consisting of one FS grade 4 (others or 1) or combinations of lesser grades exceeding limits of previous steps; able to walk without aid or rest some 300 meters.
- ❑ 5.0 - Ambulatory without aid or rest for about 200 meters; disability severe enough to impair full daily activities (e.g., to work a full day without special provisions); (Usual FS equivalents are one grade 5 alone, others 0 or 1; or combinations of lesser grades usually exceeding specifications for step 4.0).
- ❑ 5.5 - Ambulatory without aid for about 100 meters; disability severe enough to preclude full daily activities; (Usual FS equivalents are one grade 5 alone, others 0 or 1; or combination of lesser grades usually exceeding those for step 4.0).
- ❑ 6.0 - Intermittent or unilateral constant assistance (cane, crutch, brace) required to walk about 100 meters with or without resting; (Usual FS equivalents are combinations with more than two FS grade 3+).

❑ 6.5 - Constant bilateral assistance (canes, crutches, braces) required to walk about 20 meters without resting; (Usual FS equivalents are combinations with more than two FS grade 3+).

❑ 7.0 - Unable to walk beyond approximately 5 meters even with aid, essentially restricted to wheelchair; wheels self in standard wheelchair and transfers alone; up and about in wheelchair some 12 hours a day; (Usual FS equivalents are combinations with more than one FS grade 4+; very rarely pyramidal grade 5 alone).

❑ 7.5 - Unable to take more than a few steps; restricted to wheelchair; may need aid in transfer; wheels self but cannot carry on in standard wheelchair a full day; May require motorized wheelchair; (Usual FS equivalents are combinations with more than one FS grade 4+).

❑ 8.0 - Essentially restricted to bed or chair or perambulated in wheelchair, but may be out of bed itself much of the day; retains many self-care functions; generally has effective use of arms; (Usual FS equivalents are combinations, generally grade 4+ in several systems).

❑ 8.5 - Essentially restricted to bed much of day; has some effective use of arm(s); retains some self-care functions; (Usual FS equivalents are combinations, generally 4+ in several systems).

❑ 9.0 - Helpless bed patient; can communicate and eat; (Usual FS equivalents are combinations, mostly grade 4+).

❑ 9.5 - Totally helpless bed patient; unable to communicate effectively or eat/swallow; (Usual FS equivalents are combinations, almost all grade 4+).

❑ 10.0 - Death due to MS.

---

*Excludes cerebral function grade 1.

Note 1: EDSS steps 1.0 to 4.5 refer to patients who are fully ambulatory and the precise step number is defined by the Functional System score(s). EDSS steps 5.0 to 9.5 are defined by the impairment to ambulation and usual equivalents in Functional Systems scores are provided.

Note 2: EDSS should not change by 1.0 step unless there is a change in the same direction of at least one step in at least one FS.

Sources: Kurtzke JF. Rating neurologic impairment in multiple sclerosis: an expanded disability status scale (EDSS). Neurology. 1983 Nov;33(11):1444-52.

Haber A, LaRocca NG. eds. Minimal Record of Disability for multiple sclerosis. New York: National Multiple Sclerosis Society; 1985.
