# Supplementary figures and images for: A novel ABCD1 gene mutation causes adrenomyeloneuropathy presenting with spastic paraplegia: A case report
Source: Medicine (Baltimore). 2024 Apr 19;103(16):e37874. doi: 10.1097/MD.0000000000037874 (PMC11029984; doi:10.1097/MD.0000000000037874)

**Supplementary Figure S1.** Head MRI, cervical spine MRI and thoracic spine MRI.

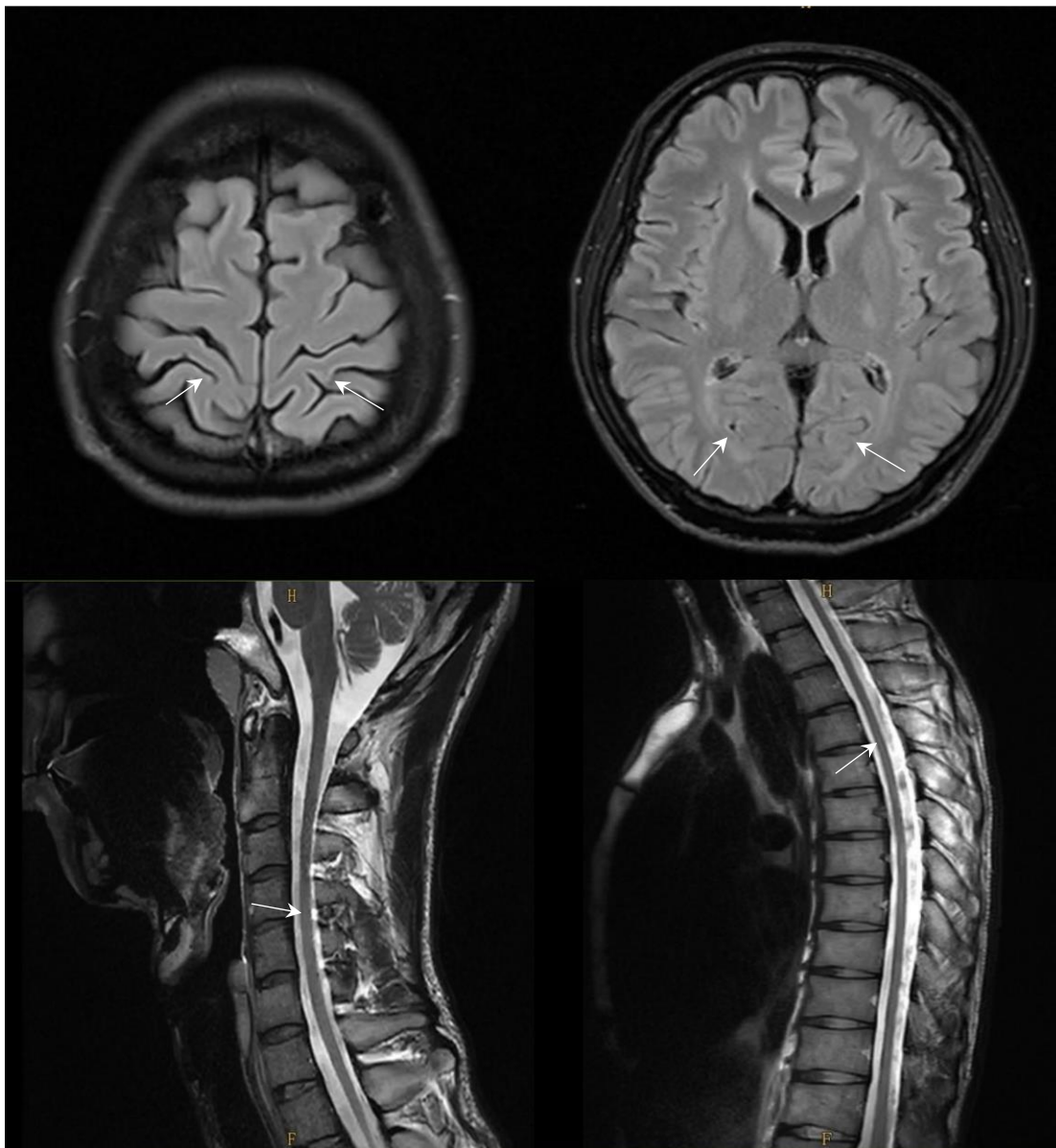

Supplement: Supplementary file 3 [file medi-103-e37874-s003.pdf]
