# Supplementary material for: A novel ABCD1 gene mutation causes adrenomyeloneuropathy presenting with spastic paraplegia: A case report
Source: Medicine (Baltimore). 2024 Apr 19;103(16):e37874. doi: 10.1097/MD.0000000000037874 (PMC11029984; doi:10.1097/MD.0000000000037874)
Supplement: Supplementary file 4 [file medi-103-e37874-s004.docx]

| **Abbreviation** | **Test Result** | **Unit** |  | **Normal Range** |
| --- | --- | --- | --- | --- |
| C10:0 | 0.06 | μmol/L | ↓ | 0.10-5.00 |
| C12:0 | 2.91 | μmol/L |  | 1.20-20.00 |
| C14:1 | 1.41 | μmol/L |  | 0.30-10.00 |
| C14:0 | 38.46 | μmol/L |  | 16.00-100.00 |
| C16:1 | 91.93 | μmol/L |  | 40.00-295.00 |
| C16:0 | 1387.01 | μmol/L |  | 860.00-2000.00 |
| C18:3 | 17.35 | μmol/L |  | 9.00-85.00 |
| C18:2 | 2155.55 | μmol/L |  | 1400.00-2810.00 |
| C18:1 | 447.44 | μmol/L |  | 400.00-1950.00 |
| C18:0 | 489.93 | μmol/L |  | 280.00-650.00 |
| C20:4 | 498.14 | μmol/L |  | 260.00-700.00 |
| C20:3 | 64.38 | μmol/L |  | 30.00-100.00 |
| C20:2 | 12.16 | μmol/L |  | 9.00-28.00 |
| C20:1 | 3.71 | μmol/L | ↓ | 6.00-27.00 |
| C20:0 | 7.04 | μmol/L | ↓ | 8.00-20.00 |
| C22:6 | 117.83 | μmol/L |  | 30.00-250.00 |
| C22:1 | 1.81 | μmol/L |  | 1.00-8.00 |
| C22:0 | 24.63 | μmol/L | ↓ | 26.00-82.00 |
| C24:1 | 29.91 | μmol/L |  | 25.00-120.00 |
| C24:0 | 62.30 | μmol/L | ↑ | 19.00-55.00 |
| C26:0 | 1.62 | μmol/L | ↑ | 0.30-0.70 |
| C24/C22 | 2.53 |  | ↑ | 0.00-0.94 |
| C26/C22 | 0.066 |  | ↑ | 0.000-0.018 |

**Supplementary Table S2.** Plasma fatty acid profile test report.
